# Evidence for Three Subgroups of Female *FMR1* Premutation Carriers Defined by Distinct Neuropsychiatric Features: A Pilot Study

**DOI:** 10.3389/fnint.2021.797546

**Published:** 2022-01-03

**Authors:** Lauren M. Schmitt, Kelli C. Dominick, Rui Liu, Ernest V. Pedapati, Lauren E. Ethridge, Elizabeth Smith, John A. Sweeney, Craig A. Erickson

**Affiliations:** ^1^Cincinnati Children’s Hospital Medical Center, Cincinnati, OH, United States; ^2^College of Medicine, University of Cincinnati, Cincinnati, OH, United States; ^3^Department of Psychology, University of Oklahoma, Norman, OK, United States; ^4^Department of Pediatrics, University of Oklahoma Health Sciences Center, Oklahoma City, OK, United States

**Keywords:** Fragile X premutation, cluster analysis, resting state EEG, executive function, Fragile X premutation carrier

## Abstract

Over 200 Cytosine-guanine-guanine (CGG) trinucleotide repeats in the 5′ untranslated region of the Fragile X mental retardation 1 (*FMR1*) gene results in a “full mutation,” clinically Fragile X Syndrome (FXS), whereas 55 – 200 repeats result in a “premutation.” *FMR1* premutation carriers (PMC) are at an increased risk for a range of psychiatric, neurocognitive, and physical conditions. Few studies have examined the variable expression of neuropsychiatric features in female PMCs, and whether heterogeneous presentation among female PMCs may reflect differential presentation of features in unique subgroups. In the current pilot study, we examined 41 female PMCs (ages 17–78 years) and 15 age-, sex-, and IQ-matched typically developing controls (TDC) across a battery of self-report, eye tracking, expressive language, neurocognitive, and resting state EEG measures to determine the feasibility of identifying discrete clusters. Secondly, we sought to identify the key features that distinguished these clusters of female PMCs. We found a three cluster solution using *k*-means clustering. Cluster 1 represented a psychiatric feature group (27% of our sample); cluster 2 represented a group with executive dysfunction and elevated high frequency neural oscillatory activity (32%); and cluster 3 represented a relatively unaffected group (41%). Our findings indicate the feasibility of using a data-driven approach to identify naturally occurring clusters in female PMCs using a multi-method assessment battery. CGG repeat count and its association with neuropsychiatric features differ across clusters. Together, our findings provide important insight into potential diverging pathophysiological mechanisms and risk factors for each female PMC cluster, which may ultimately help provide novel and individualized targets for treatment options.

## Introduction

Cytosine-guanine-guanine (CGG) trinucleotide repeat expansion in the 5′ untranslated region of *Fragile X mental retardation 1* (*FMR1*) gene over 200 and the consequent lack of protein product, Fragile X protein [FXP, formerly referred to as Fragile X mental retardation protein (FMRP)], results in a “full mutation,” clinically Fragile X Syndrome (FXS), whereas 55–200 repeats results in what is called a “premutation” (Pieretti et al., 1991; Verkerk et al., 1991). *FMR1* premutation carriers (PMCs) have reduced production of FXP (Kenneson et al., 2001) and are at an increased a risk for a range of psychiatric, neurocognitive, and physical conditions that differ from those associated with full mutation FXS (Crawford et al., 2001). Recently, the universal term of Fragile X Premutation Associated Conditions (FXPAC; Johnson et al., 2020) has been proposed to describe any behavioral and medical health symptoms associated with PMCs. FXPAC includes Fragile X-associated Primary Ovarian Insufficient (FXPOI) in females (Murray, 2000) and Fragile X-Associated Tremor/Ataxia Syndrome (FXTAS) in older males and some older females (Hagerman and Hagerman, 2021) as well as other broader commonly associated symptoms under the classification Fragile X-Associated Neuropsychiatric Disorders (FXAND; Hagerman et al., 2018; Cabal-Herrera et al., 2020; Santos et al., 2020).

Premutation status is approximately twice as likely to occur in females than males, highlighting the critical importance in better understanding neuropsychiatric features in female PMCs (Hunter et al., 2014). Previous studies have documented both intact and impaired executive function, social processing, and psychiatric features of depression and anxiety in female PMCs without FXTAS relative to age- and sex-matched typically-developing controls (TDCs) (Bennetto et al., 2001; Hessl et al., 2001; Loesch et al., 2003; Ennis et al., 2006; Allen et al., 2007; Hunter et al., 2008a,b; Rodriguez-Revenga et al., 2008; Roberts et al., 2009b,^2016^; Seltzer et al., 2012; Yang et al., 2013; Kraan et al., 2014; Mailick et al., 2014; Wheeler et al., 2014; Shelton et al., 2016; Klusek et al., 2018b; Nayar et al., 2019; Winston et al., 2020). Severity of executive dysfunction (Hunter et al., 2008b; Goodrich-Hunsaker et al., 2011a,b; Klusek et al., 2020), psychiatric symptoms (Allen et al., 2007; Roberts et al., 2009a; Seltzer et al., 2012), and social-communication differences (Schneider et al., 2016; Klusek et al., 2018a; Maltman et al., 2021) have linear and curvilinear associations with increased CGG repeat count in female PMCs. Although these findings may help account for variability in neuropsychiatric features, few studies have explored whether heterogeneous presentation among female PMCs may reflect differential presentation of these features in unique subgroups.

Two recent studies have applied clustering techniques to help identify potential subgroups of female PMCs and co-occurrence of clinical features. For example, Allen et al. (2020) reported female PMCs fell into one of eight clusters based on their self-reported medical and/or mental health diagnoses. More recently, Maltman et al. (2021) used a battery of clinical-behavioral, social-cognitive, and executive function measures to identify three clusters of female PMCs. Profile 1 whose scores across measures were at mean for PMC group; Profile 2 who demonstrated elevations in psychiatric symptoms, features associated with the broad autism phenotype, and atypical speech patterns; and Profile 3 whose scores across domains were elevated compared to the PMC mean, including in self-reported executive dysfunction. A major strength of Maltman’s work was its use of multimethod, highly quantitative data comprised of standardized self-report, informant-report, and performance-based measures, thus providing a more comprehensive approach to define clinically-meaningful clusters of female PMCs.

However, a major limitation of these previous studies was their lack of biologically-linked measures to define clusters (Budimirovic et al., 2017). A bottom-up approach to identify naturally occurring clusters of co-occurring neuropsychiatric features in female PMCs may be an important step in further parsing variability within the group, ultimately offering better insight into understanding underlying pathology and developing better screening, diagnostic, and treatment planning for this unique population. The present study aimed to build on Maltman et al. (2021) by determining the feasibility of identifying discrete clusters of female PMCs defined by neuropsychiatric features using multimodal data including self-report, eye tracking, expressive language, neurocognitive, and resting state EEG measures. Secondly, we sought to identify the key features that distinguished these clusters of female PMCs. This strategy had been applied to Tuberous Sclerosis Complex Associated Neuropsychiatric Disorders (TAND) in a small pilot sample (*n* = 56; Leclezio et al., 2018), and replicated in a slightly larger sample (*n* = 81; de Vries et al., 2020). Last, we explored whether neuropsychiatric features were differentially related to CGG repeat count within identified clusters.

## Materials and Methods

Forty-one females with PMC status between 17 and 78 years and 15 healthy TDC participants age-, IQ-, and sex matched to PMC participants were included in the study (Table 1). Only females were included in this study to control for biological sex, and to ensure feasible ascertainment. PMC status (50–200 CGG repeats) was confirmed via genetic analysis or medical record review. Potential PMC participants were asked to report any prior diagnosis of FXTAS or Parkinsonism, and were excluded if they endorsed such symptoms. Female PMCs were recruited through the Cincinnati Fragile X Research and Treatment Center, and had a child, grandchild, or sibling with FXS. TDC participants were excluded if they had any known familial history of FXS and/or were taking any medications known to affect electrophysiological measures. TDC participants were recruited through self-referral via IRB-approved advertisements on websites, electronic email blasts, or social media. Two PMCs were taking benzodiazepines and two were taking anticonvulsants at the time of testing (Supplementary Table 1), which is known to affect electrophysiological measures. Their data was included in analyses as findings did not substantively differ when excluded. All participants or their legal guardians provided informed written consent according to the Declaration of Helsinki. The local Institutional Review Board approved the study.

**TABLE 1:** Demographic data of female premutation carriers (PMC) and typically developing controls (TDC) participants.

		PMC (*N* = 41)	TDC (*N* = 15)	*P*-value
AGE		50.0 (11.8)	42.8 (12.9)	0.054
CGG		95.1 (18.3)	–	–
**IQ**				
	Full scale standard score	98.1 (11.6)	100.4 (9.9)	0.504
	Full scale deviation score	97.1 (10.8)	99.5 (8.2)	0.443
	Non-verbal *z*-score	−0.39 (1.1)	0.08 (0.7)	0.131
	Verbal *z*-score	0.00 (0.49)	−0.15 (0.6)	0.326

### Measures

#### Blood Collection

Premutation status was confirmed by *FMR1* polymerase chain reaction (PCR) with quantification of allele-specific CGG repeat length via review of medical records or through new blood collection. Blood samples were obtained from PMC participants only. Testing for *FMR1* gene CGG expansion and gene methylation was conducted at Rush University at the laboratory of Dr. Elizabeth Berry-Kravis. Six PMC participants did not complete blood draws as part of the current study. We did not include their CGG repeat count in our analysis as PCR results were not from the same laboratory.

#### Neuropsychiatric Measures

##### Resting State Electrophysiology

The EEG data was collected with 128 lead channels referenced to Cz using EGI NetAmp400 (EGI, Eugene, OR) with hydrocel nets. Consistent with our prior studies (Wang et al., 2017; Pedapati et al., 2021; Smith et al., 2021), 5-min of continuous EEG recording was obtained while participants viewed a standard silent movie to facilitate compliance and data acquisition. Data was average referenced and artifacts related to muscular, cardiac and ocular activity were removed using the ICA toolbox in EEGLAB in Matlab (Pedapati et al., 2021; Smith et al., 2021). Additional details regarding EEG collection methodology can be found in Supplementary Material 2.

##### Psychiatric Symptoms

All participants completed via the Beck Depression Inventory, Second Edition (BDI-II; Beck et al., 1996) to measure the presence and severity of depression symptoms, and the Anxiety Sensitivity Index (ASI; Reiss et al., 1986) to assess reactions experienced during anxiety-related situations. Raw scores from self-report psychiatric measures were used for statistical analysis.

##### Executive Function

Participants completed the computerized Kiddie Test of Attentional Performance (KiTAP). KiTAP examines executive functioning over multiple domains, including processing speed, cognitive flexibility, and behavioral response inhibition. Raw scores were used for statistical analysis. Though KiTAP was originally designed and normalized on children, it has been validated for use in adults with full mutation FXS (Knox et al., 2012), and raw scores have been used to compare adult control and non-control samples and in relation to EEG measures elsewhere (Bestmann et al., 2019). The NIH Cognitive Toolbox was not available at start of the study, but has since shown convergent validity across tasks in an adult sample (Hessl et al., 2016).

##### Social Attention

Eye tracking data were collected using a Tobii (Stockholm, Sweden) T120 infrared binocular eye tracker with sampling at a rate of 120 Hz. Participants completed one emotional face paradigm and one social interest paradigm. Data collection and analysis methods are described in detail elsewhere (Reisinger et al., 2019). Briefly, the emotional face paradigm consisted of colored photographs of adult human faces (equal numbers of males and females) from the NimStim Face Stimulus Set showing a calm, happy, or fearful facial expression (Farzin et al., 2009, 2011). We computed the proportion of looking time by dividing the looking time to the region of interest (e.g., eyes) by the total looking time to face. For the social interest paradigm (Hong et al., 2019), three silent 20 s side-by-side videos were presented with a social scene on one half of the screen and a geometric pattern video on the other half. The side of the social scene video was switched after each 20-s segment. Social scene preference ratio was calculated by dividing the time spent viewing the social scene videos by the total time spent viewing the social scene or geometric pattern videos.

##### Expressive Language

The Expressive Language Sampling Task (ELS) was completed by all participants (Abbeduto et al., 1995). This task assesses expressive language in a real-world, functional context by allowing participants to spontaneously create a narrative of their own while viewing a picture book. ELS has been validated for use in adults with FXS and published in an adult TDC sample (Berry-Kravis et al., 2013; Shaffer et al., 2020). Syntactic complexity, lexical diversity, talkativeness, fluency, and intelligibility were evaluated (for details regarding ELS scoring see Kover et al., 2012).

### Statistical Analysis

First, we conducted separate univariate ANOVAs for each variable with the between subjects’ factor group to examine whether female PMCs differed from matched controls. Age and IQ were evaluated as covariates, but neither significantly altered results and thus were not included in final analyses.

**FIGURE 1:**
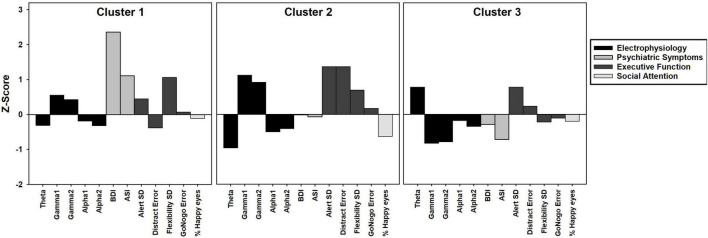
Bar graph depicting k-means cluster analysis results of three clusters of female PMC group. *Z*-scores based on means and standard deviations from TDC group. Positive *z*-scores indicate higher values than TDC, negative *z*-scores indicate lower values than TDC.

Cluster analysis was completed to identify distinct subgroups of female PMCs in SPSS version 24. *K*-means cluster analysis identifies non-hierarchical, non-overlapping clusters with the lowest within-cluster variance and the highest between-cluster variance. In order to identify the optimal number of clusters, we used the dendrogram with all possible variables and identified a three cluster solution. The optimal k was verified with the elbow point of a least-squares fit line plotted across the cluster validity index. Because k-means clustering algorithm produces round clusters, it is critical to standardize data to improve good quality clusters and improve the accuracy of the clustering algorithm (Mohamad and Usman, 2013), thus all variables were normalized (*z*-score) based on means and standard deviations of our TDC sample prior to cluster analysis (Table 2). Due to the high number of variables assessed (*n* = 45), we performed a data reduction step that was both statistically- and clinically-guided. First, we examined all variables that demonstrated significant or marginal findings (*p*’s < 0.10 and/or η^2^’s > 0.05) from between-group ANOVAs (*m* = 8 variables selected). When more than one variable from a specific KiTAP subtest met this criteria, we removed the variables with the lower η^2^ (*m* = 6). Next, we added whole brain resting state relative power in frequency bands known to be relevant to *FMR1* [alpha1, alpha2, theta, gamma1, gamma2; Wang et al., 2017; Lovelace et al., 2018; Goswami et al., 2019); *m* = 11)]. We also included the proportion of time looking at happy eyes as most likely to differentiate between subgroups based on previous reports [(Klusek et al., 2017); *m* = 12)]. Last, clinical expert opinion reviewed variables and identified additional variables of ASI total believed to be relevant to the female PMC phenotype (CE, KD, and LS). A total of 12 variables were used for k-means cluster analysis (Figure 1 and Table 3). The CGG repeat count and ELS were excluded from cluster analysis due to missing data from six and eleven participants, respectively. However, secondary analyses using univariate ANOVAs compared these features among identified clusters.

**TABLE 2:** Neuropsychiatric feature comparison between PMC and TDC participants.

		PMC (*N* = 41)	TDC (*N* = 15)	*F*-value	Uncorrected *P*-value	E.S.
BDI		11.6 (9.5)	6.5 (7.0)	3.19	0.080	0.060
ASI		15.7 (8.8)	14.4 (7.5)	0.22	0.643	0.005
**KITAP**						
Alert	Mean	350.9 (90.5)	397.7 (59.3)	4.03	0.050	0.071
	*SD*	61.5 (31.7)	40.3 (18.5)	5.90	0.019*	0.100
	Correct	30.0 (0.0)	29.3 (2.6)	2.75	0.103	0.049
Distract^†^	Mean	493.0 (99.0)	487.9 (81.3)	0.03	0.858	0.001
	*SD*	92.8 (11.1)	57.9 (18.2)	2.69	0.107	0.048
	Correct	19.2 (1.7)	19.7 (1.0)	1.22	0.274	0.023
	Error	2.8 (3.8)	0.9 (1.4)	3.50	0.067	0.062
Flex	Mean	756.7 (188.4)	670.0 (122.9)	2.72	0.105	0.049
	*SD*	192.8 (107.4)	145.3 (53.3)	2.69	0.108	0.048
	Correct	44.2 (6.7)	47.8 (0.6)	4.22	0.045*	0.074
	Error	1.6 (3.0)	0.1 (0.4)	3.64	0.062	0.064
Go/no-go	Mean	459.1 (90.2)	444.2 (66.6)	0.32	0.576	0.006
	*SD*	78.6 (32.0)	68.4 (36.2)	0.97	0.330	0.018
	Correct	19.9 (0.4)	20.0 (0.0)	0.64	0.429	0.012
	Error	0.6 (1.0)	0.2 (0.4)	2.87	0.096	0.052
**ELS**						
	Syntactic complexity	11.6 (2.6)	12.2 (1.9)	0.60	0.443	0.014
	Lexical diversity	149.2 (33.4)	172.3 (28.5)	4.95	0.032*	0.108
	% unintelligibility	1.0 (0.04)	1.0 (0.01)	1.08	0.306	0.026
	Talkativeness	10.9 (3.4)	11.1 (2.1)	0.07	0.787	0.002
	% dysfluency	0.2 (0.1)	0.3 (0.2)	2.30	0.137	0.053
**Eye tracking**						
Faces	Happy eyes%	48.1 (21.1)	51.2 (23.0)	0.21	0.641	0.004
	Calm eyes%	52.3 (21.7)	50.2 (25.1)	0.09	0.771	<0.001
	Fear eyes%	51.1 (20.4)	51.3 (22.6)	0.001	0.973	0.002
	All eyes%	50.5 (20.2)	50.9 (22.6)	0.005	0.946	<0.001
Social	Social%	63.4 (25.4)	61.6 (29.3)	0.112	0.739	0.002

Values for PMC and TDC presented as mean (standard deviation) and * indicates p-values < 0.05. ES, effect size, partial η^2^; BDI, Beck Depression Inventory; ASI, Anxiety Sensitivity Index; ^†^values for distractibility subtest only provided for trials in which distraction was present.

**TABLE 3:** *Z*-scores (based on TDC data) of variables chosen for *k*-mean cluster analysis.

		Cluster				
Domain		1	2	3	*F*	*p*-value
Electrophysiology	Theta	−0.31	−0.96	0.78	6.42	0.008*
	Gamma1	0.55	1.12	−0.83	10.33	0.001*
	Gamma2	0.43	0.92	−0.79	14.67	<0.001*
	Alpha1	−0.19	−0.50	−0.18	1.10	0.356
	Alpha2	−0.32	−0.41	−0.35	0.07	0.935
Psychiatric	BDI	2.36	−0.01	−0.29	26.67	<0.001*
symptoms	ASI	1.11	−0.07	−0.72	5.07	0.018*
Executive	Alert *SD*	0.45	1.37	0.78	1.52	0.246
function	Distract error	−0.38	1.37	0.23	4.80	0.021*
	Flexibility *SD*	1.06	0.70	−0.22	2.01	0.159
	Go/No-go error	0.07	0.17	−0.11	0.135	0.874
Social attention	% happy eyes	−0.11	−0.63	−0.20	0.450	0.645

Scores provided for each cluster, and p-values recorded based on comparison between clusters. * indicated p-value < 0.05.

Given the large age range of our analyses (17–78 years), we also completed all analyses on a smaller subset of adults 18–55 years. However, findings were nearly identical to those performed with the larger data set, thus we report only findings from the larger complete sample. Due to descriptive nature of these group comparisons and the relatively small sample size for this pilot study, corrections for multiple comparisons were not performed.

## Results

### Premutation Carriers vs. Typically Developing Controls Group Differences

Premutation carriers and TDC groups as a whole did not differ on self-reported psychiatric symptoms or most neurocognitive testing variables (Table 2). However, female PMCs had significantly greater trial-to-trial variability of reaction time during a basic processing speed task (*F* = 5.90, *p* = 0.019) and increased errors during a flexibility task (*F* = 4.22, *p* = 0.045) on the KiTAP than TDC. During ELS, female PMCs had significantly less lexical diversity than TDC (*F* = 4.95, *p* = 0.032), suggesting the size of female PMCs’ expressive vocabularies is smaller than those of TDC. However, groups were similar on other ELS variables (*p*’s > 0.137). No significant group differences were found across eye tracking variables or in resting relative power for any frequency band (*p*’s > 0.15; Supplementary Material 2).

### Premutation Carrier Cluster Analysis

**FIGURE 2:**
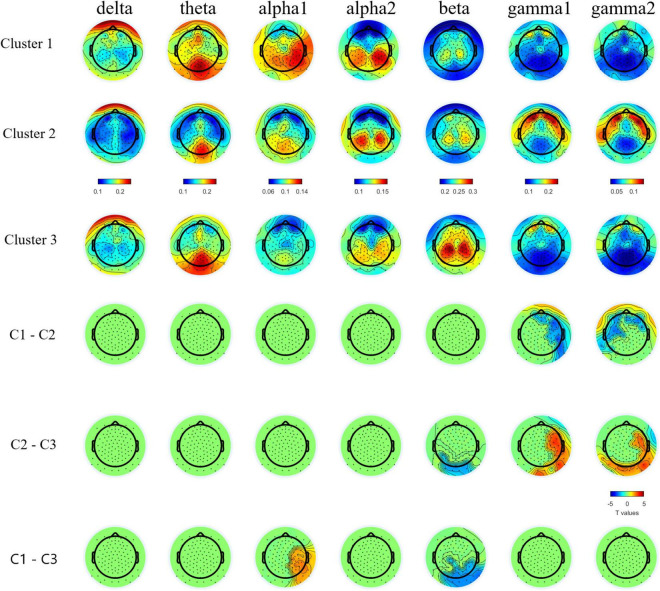
Topographic plots of group averaged relative band power in bands and topographical plots of T values from band specific pairwise group comparisons. Cluster-based permutation test detected lower relative gamma1 and gamm2 bands powers in C1 and C3 than C2, lower relative beta band power in C1 and C2 than C3, and higher relative alpha1 band power in C1 than C3. For multiple comparison cross bands, one directional family-wise alpha level was set at 0.05.

We found a three cluster solution with maximum distance between the different clusters confirmed by *F*-tests (Table 3 and Figure 1). Cluster 1 is primarily defined by elevated psychiatric symptoms, including depression and anxiety, with relatively typical electrophysiological features (*n* = 11, 27% of PMC sample). Cluster 2 is primarily defined by impaired cognitive processing in the form of executive dysfunction as well as elevated high frequency resting gamma power (*n* = 13, 32%). Cluster 3 represents a relatively unaffected group with minimal abnormalities across neuropsychiatric features (*n* = 17, 41%). Power band comparisons by clusters are available in Figure 2, and further detailed in Supplementary Figures 1–3.

To further examine our defined PMC clusters, we compared clusters on variables not included in the original cluster analysis. There was no difference in IQ or age between clusters (*F*’s < 0.39, *p*’s > 0.67; Table 4). Medication usage also did not differ between clusters (*X*^2^’s < 1.7, *p*’s > 0.42). Talkativeness during ELS differed between clusters [*F*(2,29) = 4.90, *p* = 0.02], with female PMCs in Cluster 2 being significantly more talkative than those in Cluster 3 (*t* = 3.11, *p* = 0.004). CGG repeat count also differed based on cluster membership [*F*(2,35) = 3.72, *p* = 0.04]. Female PMCs in Cluster 1 had higher CGG repeat counts compared to those in Cluster 2 (*t* = 2.42, *p* = 0.02) and Cluster 3 (*t* = 2.44, *p* = 0.02). However, Cluster 2 and 3 did not differ on repeat counts (*p* = 0.97).

**TABLE 4:** Comparison of mean (standard deviation) performance on measures across clusters.

		Cluster					
Medication		1	2	3	Chi	*p*-value	
*N* (%)	Anti-convulsant	1 (9)	1 (8)	0 (0)	1.74	0.42	
	Anti-depressant	4 (36)	4 (31)	5 (30)	0.87	0.65	
	Benzodiazepine	1 (9)	0 (0)	1 (6)	0.64	0.73	

**Domain**		**1**	**2**	**3**	***F***	***p*-value**	***Post hoc***

Demographic	Age	47.9 (15.2)	49.3 (12.1)	51.9 (8.3)	0.40	0.68	
	*IQ*	101.0 (12.7)	97.9 (11.7)	96.6 (11.4)	0.40	0.68	
CGG repeat count		108.4 (17.3)	89.8 (14.0)	90.2 (20.0)	3.72	0.04	1 vs. 2 = 0.02 1 vs. 3 = 0.02
Neurophysiology	Theta	0.162 (0.02)	0.132 (0.05)	0.188 (0.03)	8.88	0.001	1 vs. 2 = 0.05 1 vs. 3 = 0.07 2 vs. 3 < 0.001
	Gamma1	0.192 (0.04)	0.237 (0.08)	0.156 (0.03)	8.13	0.001	1 vs. 2 = 0.05 1 vs. 3 = 0.10 2 vs. 3 < 0.001
	Gamma2	0.100 (0.03)	0.128 (0.05)	0.080 (0.02)	6.79	0.003	1 vs. 2 = 0.06 1 vs. 3 = 0.15 2 vs. 3 = 0.001
	Alpha1	0.065 (0.02)	0.046 (0.01)	0.056 (0.01)	4.35	0.020	1 vs. 2 = 0.01 1 vs. 3 = 0.13 2 vs. 3 = 0.11
	Alpha2	0.048 (0.01)	0.034 (0.01)	0.041 (0.01)	4.07	0.025	1 vs. 2 = 0.01 1 vs. 3 = 0.19 2 vs. 3 = 0.10
Psychiatric	BDI	21.5 (5.7)	11.1 (9.5)	5.2 (4.8)	19.05	<0.001	1 vs. 2 = 0.001 1 vs. 3 < 0.001 2 vs. 3 = 0.03
	ASI	21.0 (8.4)	16.4 (9.5)	11.5 (6.7)	4.50	0.02	1 vs. 2 = 0.22 1 vs. 3 = 0.01 2 vs. 3 = 0.15
Expressive language sampling	Syntactic complexity	11.1 (3.0)	10.5 (1.6)	12.5 (2.8)	1.54	0.24	
	Lexical diversity	127 (33)	158 (30)	152 (36)	0.82	0.45	
	% unintelligibility	98.1 (3.9)	99.5 (0.8)	98.6 (4.3)	0.29	0.75	
	Talkativeness	11.2 (3.7)	13.6 (2.5)	0.3 (2.9)	4.90	0.02	1 vs. 2 = 0.12 1 vs. 3 = 0.17 2 vs. 3 = 0.004
	% dysfluency	25.0 (11.1)	22.8 (21.3)	15.7 (9.5)	1.37	0.27	
Executive function	Alert *SD*	56.5 (19.7)	71.5 (44.7)	57.6 (27.0)	0.86	0.43	
	Distract error	1.5 (2.1)	5.2 (5.3)	1.7 (2.0)	4.63	0.02	1 vs. 2 = 0.01 1 vs. 3 < 0.001 2 vs. 3 = 0.03
	Flexibility *SD*	238 (127)	216 (126)	143 (45)	3.38	0.04	1 vs. 2 = 0.58 1 vs. 3 = 0.02 2 vs. 3 = 0.06
	Go/no-go error	0.64 (0.92)	1.00 (1.16)	0.25 (0.78)	2.23	0.12	
Social attention	% happy eyes	45.3 (20.6)	52.0 (17.5)	46.9 (24.4)	0.35	0.71	

*Post hoc comparisons provided for any significant group comparisons.*

We also examined difference between clusters when using categorized repeat count based on previous papers and as recommended by the American College of Medical Genetics (Sherman et al., 2005; low: 61–80 repeats, mid: 81–100 repeats, and high: 100–199 repeats). Using a linear-by-linear association test, we found a marginally significant relationship between cluster and repeat count category (LxL^2^ = 3.03, *p* = 0.08). Specifically, Cluster 1 had more individuals in the High CGG repeat category (67%) compared to Cluster 2 (17%) and Cluster 3 (21%). PMCs considered to have mid-range CGG repeat count composed 50% of Cluster 2 and Cluster 3 each, but only 22% of Cluster 1.

### Correlations Within Clusters

**FIGURE 3:**
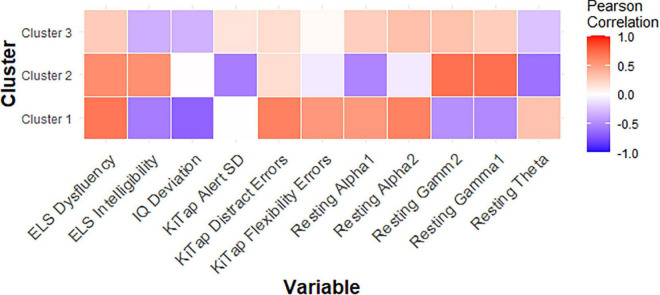
Correlation matrix showing relationships between key variables and CGG repeat count for each PMC cluster.

Among female PMCs in Cluster 1, we found higher CGG repeat count was significantly related to fewer correct trials during the KiTAP Distractor subtask (*r* = −0.75, *p* = 0.02; Figure 3). We also found higher gamma1 and gamma2 resting power were associated with more correct Distractor trials (gamma1: *r* = 0.75, *p* = 0.01; gamma2: *r* = 0.76, *p* = 0.01).

In female PMCs classified as Cluster 2, higher CGG repeat count was associated with lower theta power (*r* = −0.61, *p* = 0.04), but higher gamma1 (*r* = 0.72, *p* = 0.01) and gamma2 (*r* = 0.71, *p* = 0.01) power. Lower theta also was associated with greater proportion of time viewing social scenes during the dynamic eye tracking task (*r* = −0.56, *p* = 0.04), but higher gamma also was associated with greater proportion of looking at calm eyes during static face task (gamma1: *r* = 0.56, *p* = 0.04; gamma2: *r* = 0.56, *p* = 0.04) in Cluster 2.

Last, among female PMCs in Cluster 3, elevated theta (*r* = 0.54, *p* = 0.03) but reduced gamma1 (*r* = −0.49, *p* = 0.04) and reduced gamma2 (*r* = −0.66, *p* = 0.004) were associated with greater proportion of time looking at eyes across emotions. Elevated theta also was related to greater number of errors during KiTAP Flexibility in Cluster 3 (*r* = 0.51, *p* = 0.04).

## Discussion

In this pilot cluster analysis study of female Fragile X premutation carriers, we identified three distinct subgroups defined by neuropsychiatric features: a psychiatric feature group, an executive dysfunction and altered electrophysiology group, and an unaffected group. Our findings indicate the feasibility of using a data-driven approach to parsing heterogeneity in a biologically-meaningful way and identifying naturally-occurring clusters in female PMCs using a multi-method assessment battery consisting of self-report, performance-based, and electrophysiological measures. Together, these findings offer novel insights into different neuropsychiatric profiles in this unique population, which may make a critical difference in understanding pathophysiological mechanisms and identifying targeted interventions for female PMCs.

### Preserved Cognitive, Social, Neurophysiological Functioning at Group-Level

Consistent with several (Bennetto et al., 2001; Loesch et al., 2003; Bailey et al., 2008a; Hunter et al., 2008b; Yang et al., 2013; Allen et al., 2020) but not all previous studies (Hunter et al., 2010; Hagerman and Hagerman, 2013; Lozano et al., 2016; Napoli et al., 2016), we found that neuropsychiatric functioning in our sample of female PMCs was relatively similar to matched controls. This finding emerged across nearly all the measures we assessed, suggesting the incomplete and variable penetrance of neuropsychiatric features in female PMCs.

### Cluster Membership

We found a three cluster solution using k-means clustering. Cluster 1 represented a psychiatric feature group (27% of our sample); Cluster 2 represented a group with executive dysfunction and elevated high frequency (gamma) neural oscillatory activity (32%); and Cluster 3 represented a relatively unaffected group (41%). These represent three clinically-meaningful clusters with good clinical face-validity (Hagerman et al., 2018) and considerable overlap with clusters recently identified by Maltman et al. (2021).

Psychiatric features and executive dysfunction have been well-documented in female PMCs; however, the majority of previous studies have examined these features independently (for review see Hagerman et al., 2018). Our study using a multimodal data collection approach provides the first indication that cognitive features associated with PMC (i.e., executive dysfunction) tend to co-occur with aberrant electrophysiological features, and define a subgroup of female PMCs separate from those who show predominantly psychiatric features. This finding supports and extends previous studies demonstrating that elevated psychiatric symptoms and executive dysfunction may be key features of separate subgroups of female PMCs (Allen et al., 2020; Maltman et al., 2021). For example Maltman et al. (2021) demonstrated that one subgroup of female PMCs was characterized by elevated mood and anxiety symptoms and a separate, smaller subgroup was characterized by elevated executive dysfunction and impaired social-cognitive abilities. It is worth noting that distinct psychiatric and cognitive clusters also were identified in TAND (Leclezio et al., 2018; de Vries et al., 2020). Thus, given our finding of a unique electrophysiological profile co-occurred with executive dysfunction in a subgroup of female PMC, it is possible that different upstream neurobiological pathologies may lead to specific clustering of co-occurring neuropsychiatric features downstream.

### Neurophysiological Findings

This is the first study to date to examine relative power during continuous resting state EEG recording in female PMCs. Given the lack of significant differences at the group level between PMC and TDC in the other neuropsychiatric features examined, it is not surprising that these groups also did not differ overall in resting state power across frequency bands. Yet, among the most consistent and replicated finding in FXS research across studies in mouse, human, and *in vivo* brain slices is network hyperexcitability, thought to be caused by an imbalance of neuronal excitation:inhibition (E:I) and to be reflected in elevated gamma band power (Ronesi et al., 2012; Ethridge et al., 2017; Wang et al., 2017; Lovelace et al., 2018; Goswami et al., 2019; Jonak et al., 2020). Thus, given the consistency of this hyperexcitability finding and its presumed causal effect of deficient FXP production (Contractor et al., 2015), electrophysiological features would be expected to be present in a subset female PMCs.

Indeed, a distinct electrophysiological profile of reduced theta and increased gamma1 and gamma2 emerged for female PMCs in Cluster 2. Increased high frequency gamma1 and gamma2 resting power has been reported in full mutation FXS (Wang et al., 2017; Pedapati et al., 2021; Smith et al., 2021). Our findings suggest a potential electrophysiological signature specific to Cluster 2 that is consistent with E:I imbalance as seen in full mutation FXS. Notably, among female PMC with gamma power at least 1SD above TDC mean, 78% were classified within Cluster 2, suggesting that elevated high frequency resting power may occur in other female PMCs outside of this subgroup, but these elevations tends to co-occur with executive dysfunction. The co-occurrence of these features implicate the need for future research to determine whether these key phenotypes relate causally.

Previous studies have reported both increased relative gamma and theta power in full mutation FXS compared to TDC (Van der Molen and Van der Molen, 2013; van der Molen et al., 2014; Wang et al., 2017; Pedapati et al., 2021). Visual inspection of Figure 1 reveals the majority of females within Cluster 2 had gamma power 1SD *above* the mean of TDC, but theta power 1SD *below* the mean of TDC. While much needs to be learned about low frequency alterations of EEG power in FXS, there is a pattern of more theta band activity that is inversely related to the high frequency background neural oscillatory activity. This theta-gamma relation is thought to represent compensation for the considerably elevated gamma band activity in FXS (Wang et al., 2017; Pedapati et al., 2021; Smith et al., 2021), which perhaps is not needed in PMC based on our findings. Alternatively, Cluster 2 may represent a subgroup of PMC who are unable to mobilize compensatory neural activity in the theta band, contributing to executive dysfunction (Wang et al., 2017; Ethridge et al., 2019; Pedapati et al., 2021). Other possibilities exist, but what is evident in our data is that aberrant gamma band activity suggesting E:I imbalance, along with altered lower frequency patterns, are present among some female PMCs as well as in full mutation FXS.

Compared to TDC, individuals in Cluster 1 (the “psychiatric feature” subgroup) did not differ in EEG relative band power distribution. In contrast, Cluster 3 (the “unaffected” subgroup) demonstrated increased theta power and reduced alpha power in comparison with TDC. Female PMCs in Cluster 3 also showed the lower gamma power compared to TDC and other PMC clusters. These findings suggest a potential compensatory mechanism in female PMCs in Cluster 3 to dampen high frequency gamma activity, which could ultimately lead to their relatively spared neuropsychiatric phenotype. Future studies with larger female PMC samples are needed to replicate and further explore these electrophysiological profiles.

### Cluster Profiles in Detail

#### Cluster 1

Female PMCs in Cluster 1 were noteworthy for the presence of elevated psychiatric symptoms and high CGG repeat counts, although these specific features did not interrelate with each other. In contrast, we found higher CGG repeat count and greater elevations in high frequency power was associated with more severe distractibility in this subgroup. Though we generally observed psychiatric features and executive dysfunction to be the predominant phenotypes of distinct subgroups, vulnerability to specific executive function deficits in Cluster 1 may result from underlying biology. This notion also could help account, in part, for our finding of a relatively preserved electrophysiological profile in female PMCs in Cluster1 since executive dysfunction was largely absent in this subgroup. Instead, environmental factors, including the stress associated with being a (grand)parent to or sibling of a child with FXS, may be important sources of emotional distress in this subgroup.

#### Cluster 2

Cluster 2 is defined by executive dysfunction and an electrophysiological profile of reduced theta and increased gamma1 and gamma2. We found that females in Cluster 2 with larger CGG repeat count had more aberrant theta and gamma power, indicating high CGG repeat count likely accounts, in part, for this electrophysiological profile. This finding suggests an important electrophysiological link to a common molecular biomarker in female PMCs that has not been previously identified. However, KiTAP variables did not relate to this electrophysiological profile or to CGG repeat count. In fact, we unexpectedly found that this subgroup had the *lowest* percentage of females in the high CGG repeat category. It is possible that executive dysfunction may be present in Cluster 2 regardless of degree of electrophysiological “deficit” or CGG repeat count, such that other biological and/or environmental determinants determine the degree of executive dysfunction. A recent study documented CGG repeat count and stress independently contributed to executive dysfunction in female PMCs (Maltman et al., 2020). In the context of our current findings, this suggests biological and environmental risk factors associated with executive dysfunction are not limited to female PMCs with high CGG repeat count. Understanding how other molecular correlations (e.g., Fragile X protein levels) and pathophysiological mechanisms underlie the unique neuropsychiatric features of Cluster 2 is a critical area to pursue in future work.

#### Cluster 3

Aside from the electrophysiological profile described for Cluster 3, this subgroup of female PMCs demonstrated relatively spared neuropsychiatric function, and thus was labeled the “unaffected” group. Given female PMCs in Cluster 3 still have underlying pathology associated with *FMR1* gene, it is possible that this cluster has developed compensatory mechanisms and/or have additional protective factors in order to be relatively spared from characteristic neuropsychiatric features. For example, individuals in Cluster 3 may utilize increased theta power to compensate for tendency toward neural hyperexcitability as seen in Cluster 2, thereby serving as a mediator for neuropsychiatric deficits. As seen in our previous work with full mutation FXS (Wang et al., 2017; Ethridge et al., 2019; Pedapati et al., 2021), enhanced theta power as a compensatory mechanism may target certain aspects of pathophysiology. However, it is not a perfect solution, and may also accompany other behavioral deficits. Therefore, although enhanced theta power may protect and/or enhance sensory and social processing pathways (e.g., time looking at eyes) in this subgroup of female PMCs, this electrophysiological signature also is associated with increased flexibility error rate on the KiTAP. Resolving the extent to which enhanced theta power plays a role in this relatively spared neuropsychiatric profile could provide critical insights into potential interventions for females PMCs in other subgroups and in full mutation FXS.

### Pathophysiological Mechanisms of Neuropsychiatric Outcomes

Current consensus in the field is that among female PMCs, genetic risk factors like CGG repeat count, Fragile X protein level, degree of toxicity resulting from CGG repeat containing *FMR1* messenger ribonucleic acid (mRNA), percent methylation, non-AUG (RAN) translation, heritable factors not related to the *FMR1* gene, and environmental factors including increased stress in family members of individuals with FXS all are thought to play a role in the heterogeneous clinical presentation of female PMCs (Hartley et al., 2012; Seltzer et al., 2012; Hall et al., 2016). For example, one important consideration is elevated levels of mRNA occur in both FXS and PMC, which is believed to rise to toxicity level with increasing CGG repeat count in individuals with PMC (Hoem et al., 2011; Qurashi et al., 2011). However, as CGG repeat count approaches that of full mutation Fragile X (>200 repeats), the *FMR1* gene becomes fully methylated, and thus mRNA levels decline. However, declining mRNA with increasing CGG repeats may be counteracted by other CGG-dependent changes in gene expression, as mid-range CGG repeat count has been associated with increased Fragile X protein production (Loesch et al., 2007; Peprah et al., 2010; Sellier and Charlet-Berguerand, 2014). However, we did not see any associations with mid-range CGG repeat via non-linear associations in the current study.

Finally, it is as important to consider the sex-specific pathways involved in neural development as well as the effects of X-inactivation in female PMC as it is in females with full mutation FXS (Alvarez-Mora et al., 2016; Hall et al., 2016). For example, neuropsychiatric profiles in male and female PMCs may differ early *in utero* and subsequently proceed among different developmental pathways. Several lines of neurodevelopmental and FXTAS research indicate the protective nature of being female, which may provide alternative compensatory mechanisms to this population (for example, see Loesch et al., 2020). Future research is needed to determine the degree to which these and other factors account for neuropsychiatric features in female PMCs.

### Limitations

The sample was limited to female PMCs and excluded male PMCs. Future studies are needed to determine whether clusters identified in the present study also are found among male PMCs. A strength of our study was only including females PMCs who had an immediate family member participate in our larger NIH-funded Fragile X Center study, thus all females had an environmental stress factor in common. Still this selection bias may have impacted results and only be specific to female PMCs who are relatives of individuals with FXS. Additionally, the current study lacked measurement of the effects of life stressors and social supports in relation to neuropsychiatric features, which have been documented in past studies (Abbeduto et al., 2004; Lewis et al., 2006; Bailey et al., 2008b; Hartley et al., 2012; Seltzer et al., 2012). The extent to which phenotypic variation and cluster membership is due to these factors needs to be considered in future studies following up on our findings. We also consider the use of a large age-range as a strength of this study as it captures clusters occurring across young, middle, and older adulthood. Although the upper age limit may be a concern for late-onset conditions including FXTAS and non-PMC age-related decline, we found no age effects in either our case-control or cluster findings. Additionally, no female PMCs had a diagnosis of FXTAS based on medical history and physician report.

The small sample size of female PMCs is a notable limitation, though for a pilot study is likely appropriate especially as we largely replicate and extend prior findings, including Maltman et al. (2021) cluster study with a larger sample. A large replication study is needed in the future to confirm cluster results. Importantly, our sample size is similar to that used in the initial feasibility study to identify clusters in TAND (Leclezio et al., 2018; de Vries et al., 2020). Additionally, using a multimodal approach to data collection, including self-report, performance-based, and electrophysiological measures, provided us with perhaps a greater signal opportunity to detect distinct patterns neuropsychiatric features within PMC participants. Our approach to variable selection method and utilization of *k*-means cluster analysis may have biased findings, which were sample dependent. Though we demonstrated the feasibility of subgrouping females PMCs into biologically-meaningful and clinically-valid clusters, more sophisticated feature selection techniques and clustering methods are needed in future studies to replicate current findings.

Approximately 40% of our PMC sample reported taking at least one psychiatric medication at the time of the study (e.g., SSRI and benzodiazepine). Yet, it is notable that psychiatric medication use was consistent across the three clusters. This suggests that some female PMCs may have been effectively treated and demonstrate reduced or subthreshold psychiatric symptoms, which could have affected cluster membership. Though four female PMCs at the time of testing were taking certain psychiatric medications may interfere with electrophysiological recordings, excluding these participant’s data from our analyses did not substantively change findings.

Specific measures, including KiTAP and ELS, previously have not been used in PMC samples, thus additional caution was taken with data interpretation. PMC group performance was relatively similar to controls, consistent with numerous studies using other measures of executive function and language, suggesting its use in the current study is appropriate. Last, we also did not obtain blood from TDC, and thus it is possible that a control participant may have had CGG repeat expansion in the “gray zone” or the premutation range.

## Conclusion

Consistent with prior reports, our overall sample of female PMCs has relatively preserved psychiatric, cognitive, social, and electrophysiological functioning compared to matched controls. However, given the X-linked nature of the disorder and variability in CGG repeat length, Fragile X protein levels, degree of mRNA toxicity, and environmental factors, variable neuropsychiatric profiles are both well-documented and expected in female PMC even if group comparisons are null. Our pilot study using a wide array of quantitative assessments to subgroup clusters demonstrated that we were able to identify three clusters of female participants with PMC status, each demonstrating a unique profile of neuropsychiatric features—a psychiatric feature group, and an executive dysfunction and elevated high frequency resting state power group, and an unaffected group. Together, our findings implicate the feasibility of using multimodal data to identify subgroups of female PMCs that are clinically-meaningful and face-valid, with the promise of providing important insight into potential diverging pathophysiological mechanisms and risk factors for each cluster. Future studies with larger samples are warranted to replicate cluster findings and expand on molecular correlates in order to improve our understanding of illness mechanisms that in the longer term may provide novel and individualized targets for treatment options.

## Data Availability Statement

The datasets presented in this study can be found in online repositories. The names of the repository/repositories and accession number(s) can be found below: NIMH Data Archive.

## Ethics Statement

The studies involving human participants were reviewed and approved by Cincinnati Children’s Hospital Medical Center. Written informed consent to participate in this study was provided by the participants’ legal guardian/next of kin.

## Author Contributions

LS analyzed and interpreted data and wrote full draft of the manuscript. KD and ES assisted with data interpretation and manuscript. RL, LE, and EP analyzed and interpreted resting EEG findings. JS and CE participated in the conceptualization of the study and edited the manuscript. All authors contributed to the article and approved the submitted version.

## Conflict of Interest

The authors declare that the research was conducted in the absence of any commercial or financial relationships that could be construed as a potential conflict of interest.

## Publisher’s Note

All claims expressed in this article are solely those of the authors and do not necessarily represent those of their affiliated organizations, or those of the publisher, the editors and the reviewers. Any product that may be evaluated in this article, or claim that may be made by its manufacturer, is not guaranteed or endorsed by the publisher.
