# Supplementary material for: Evidence for Three Subgroups of Female FMR1 Premutation Carriers Defined by Distinct Neuropsychiatric Features: A Pilot Study
Source: Front Integr Neurosci. 2022 Jan 3;15:797546. doi: 10.3389/fnint.2021.797546 (PMC8763356; doi:10.3389/fnint.2021.797546)
Supplement: Supplementary file 1 [file Data_Sheet_1.docx]

**Supplementary Material**

Supplemental Table 1. Medication information for PMC and TDC participants. Information was unavailable for two PMC and one TDC participants.

| **Psychotropic Medications** | **PMC** | **TDC** |
| --- | --- | --- |
| **Multiple Medications** | 17 (43.6%) | 5 (19.2%) |
| **Prescribed Psychiatric Medications** | 16 (41%) | 4 (15.4%) |
| SSRI | 10 (25.6%) | 2 (7.7%) |
| Other Antidepressants | 8 (20.5%) | 3 (11.5%) |
| Benzodiazepines | 2 (5.1%) | 0 |
| Anticonvulsant | 2 (5.1%) | 0 |
| Stimulant | 1 (2.6%) | 0 |
| **Hormone Medication** | 10 (25.6%) | 10 (38.5%) |
| **Hypertension Medication** | 10 (25.6%) | 3 (11.5%) |
| **Cholesterol Medication** | 6 (15.4%) | 1 (3.8%) |
| **Antihyperglycemic Medication** | 4 (10.3%) | 0 |
| **Proton Pump Inhibitors for GERD** | 2 (5.1%) | 4 (15.4%) |
| **Opoid analgesic** | 1 (2.6%) | 0 |
| **Antibiotics** | 2 (5.1%) | 1 (3.8%) |
| **Allergy** | 0 | 2 (7.7%) |

Supplementary Material 2

EEG Data description: EGI 128-lead HydroCel Net was utilized to collect resting state scalp EEG from 56 subjects participated in this project. To increase the usability of EEG data that represent brain activities for analysis, an EEG research coordinator preprocessed multi-dimensional raw EEG using an integrated GUI developed from EEGLAB (Delorme and Makeig 2004), including 1) bandpass filtering, 2) segment rejection, 3) channel interpolation, 4) independent components analysis to remove additive electric line noise, facial muscle activities and heart rate effects, and 5) current source density to minimize volume conduction. Additionally, 20 channels of preprocessed time series which are recorded from electrodes attached to the neck and draw are dropped from further analysis.

Methodology: Relative band power is the band accumulation of averaged relative spectrum over trials. Seven fixed bands of interest are estimated for each selected channel: delta (2-3.5 Hz), theta (4-7.5 Hz), alpha1 (8-10 Hz), alpha2 (10-13.5 Hz), beta (15-30 Hz), gamma1 (30.5-55 Hz) and gamma2 (65-80 Hz). Relative band power is compared by band per (6) pair of conditions (PMC C1/C2/C3 and TDC) below. Due to the high spatial-dimension nature of EEG data, a nonparametric spatial-cluster based permutation test (Sassenhagen and Draschkow 2018) was applied for each band cluster formation at the electrode level. Seven-band multiple comparisons are Bonferroni corrected with one-directional $\alpha$=0.05.

Results: For all condition pairwise comparisons, the topographical representations of group averaged relative power distribution are in the top two rows, and the T-statistics that constructing the significant cluster(s) of electrodes are shown in the bottom two rows (as Supplemental Figures 1-4). In detail, for an example of PMC Cluster 1 (C1) and Cluster 2 (C2), clusters in alpha1 and alpha2 bands are detected significantly higher in PMC C1 than in C2, clusters in the gamma1 and gamma2 bands are detected as lower in PMC C1 than in PMC C2. Overall band-specific existence of significant clusters is summarized in Supplementary Table 2 (no pairwise multiple comparison correction applied).

Supplementary Table 2. Summary of group-level relative power comparison per band.

| ^Delta^ | ^Theta^ | ^Alpha1^ | ^Alpha2^ | ^Beta^ | ^Gamma1^ | ^Gamma2^ |
| --- | --- | --- | --- | --- | --- | --- |
| ^C3>C1^ | ^C3>C1^ | ^C1>C2^ | ^C1>C2^ | ^C1>C3^ | ^C2>C1^ | ^C2>C1^ |
|  | ^C3>C2^ | ^C1>C3^ | ^C3>C2^ | ^C2>C3^ | ^C1>C3^ | ^C1>C3^ |
|  |  |  | ^TDC>C2^ | ^C1>TDC^ | ^C2>C3^ | ^C2>C3^ |
|  |  |  | ^TDC>C3^ | ^TDC>C3^ | ^C2>TDC^ | ^C2>TDC^ |
|  |  |  |  |  | ^TDC>C3^ | ^TDC>C3^ |

Supplementary Figure 1. PMC versus TDC group comparison on resting state EEG power across frequency bands.
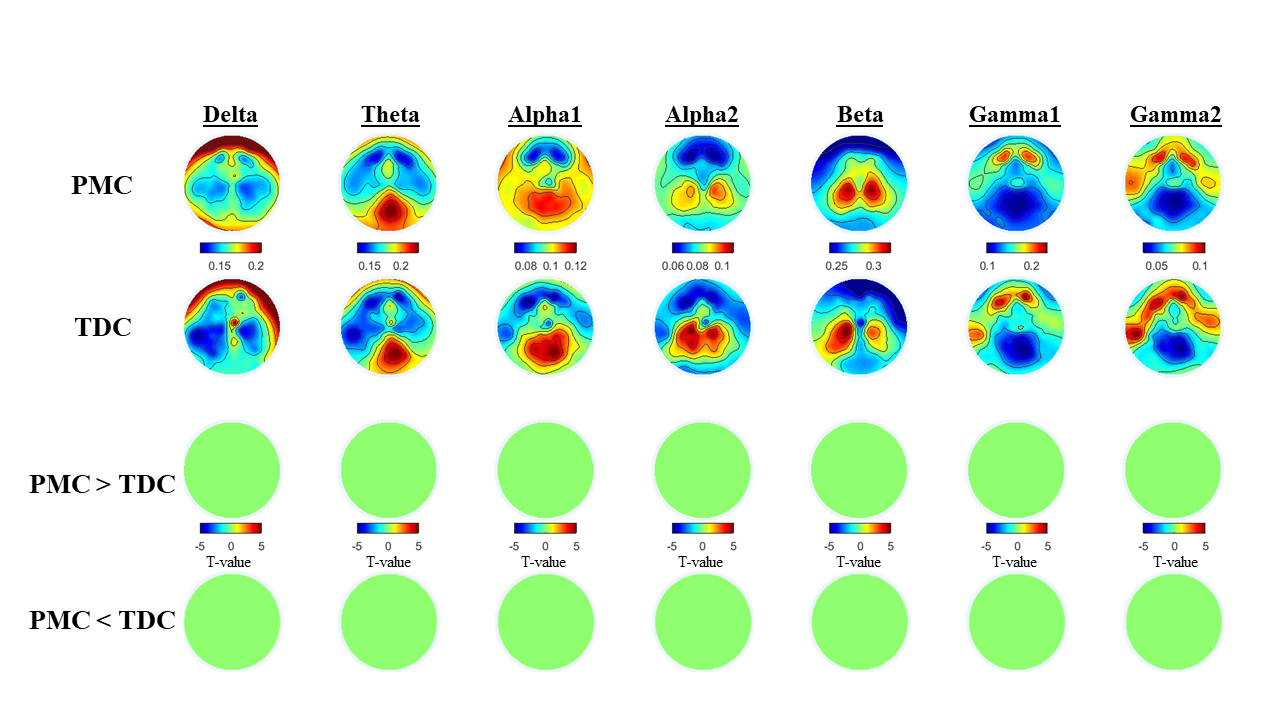


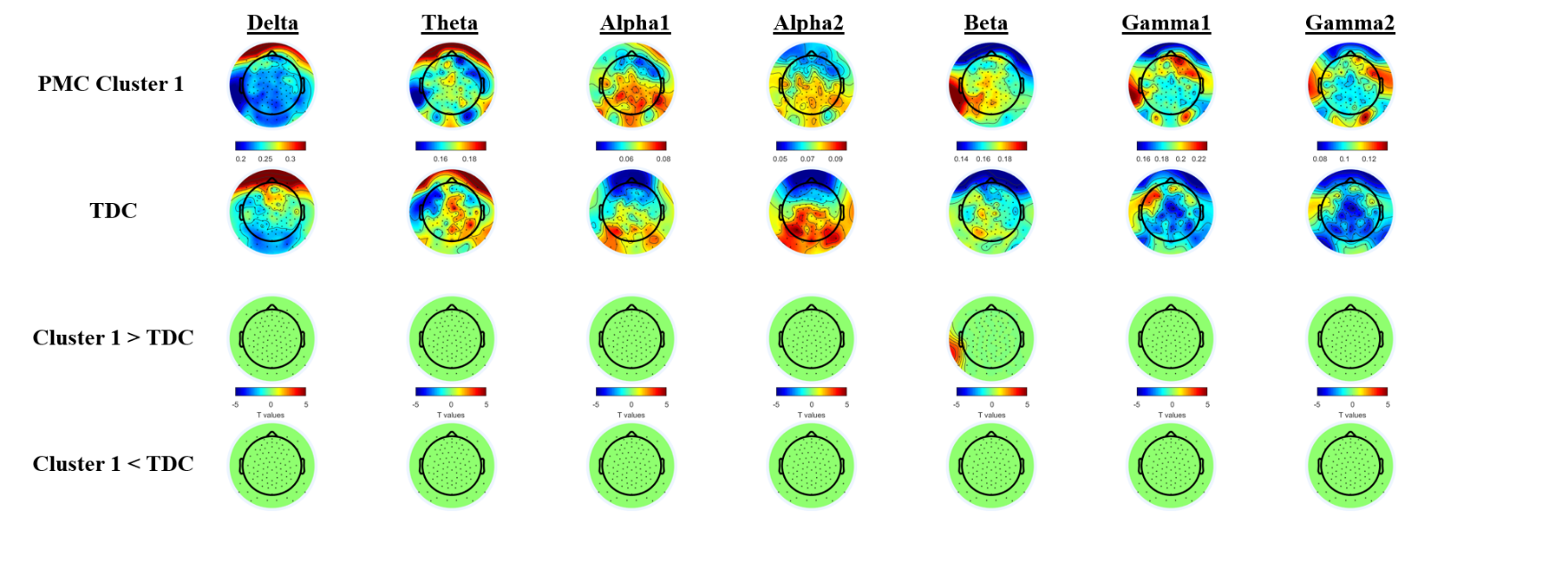
Supplementary Figure 2. PMC Cluster 1 versus TDC group comparison on resting state EEG power across frequency band.


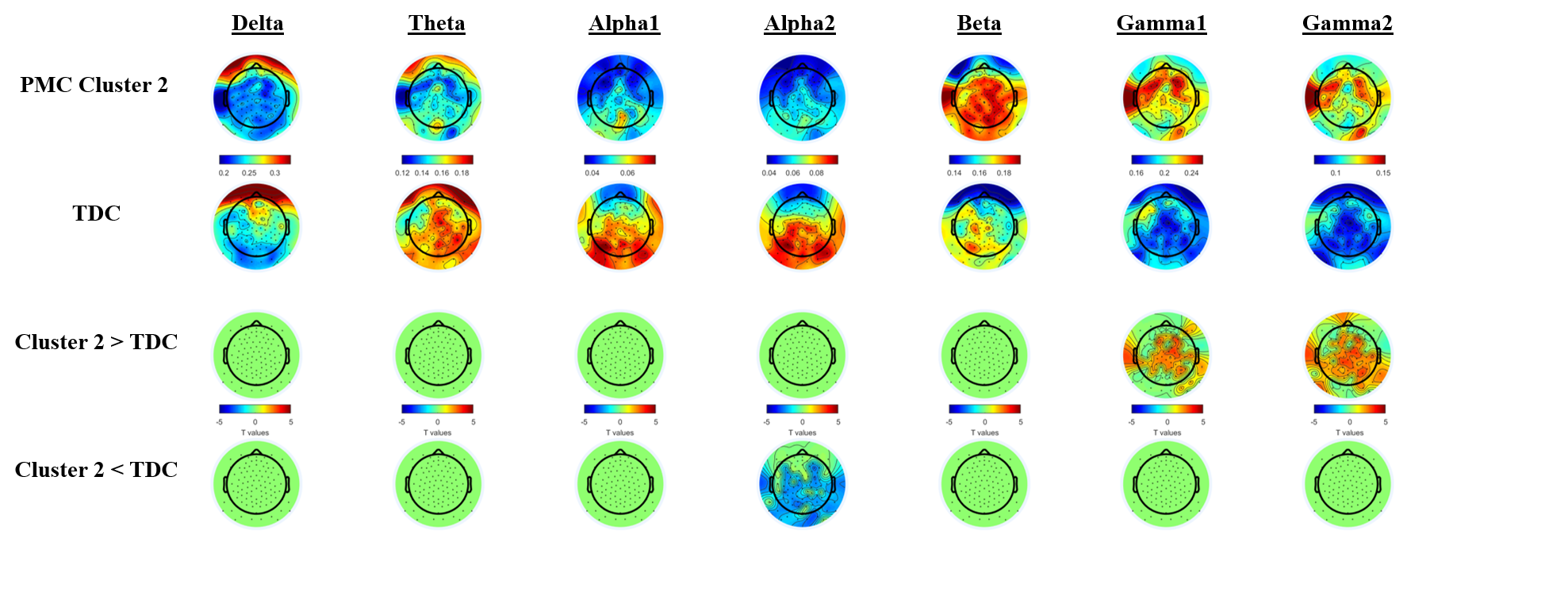
Supplementary Figure 3. PMC Cluster 2 versus TDC group comparison on resting state EEG power across frequency band

Supplementary Figure 3. PMC Cluster 3 versus TDC group comparison on resting state EEG power across frequency band


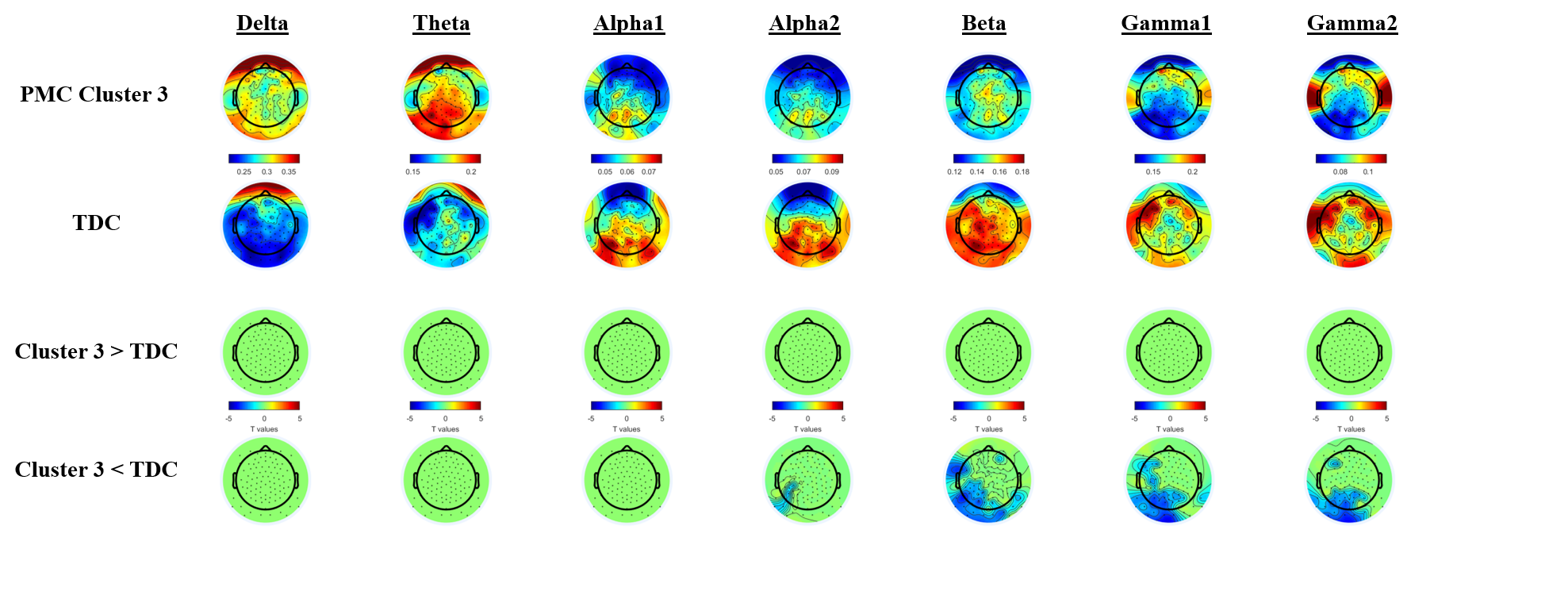


Reference

Delorme A and Makeig S (2004) EEGLAB: an open-source toolbox for analysis of single-trial EEG dynamics, Journal of Neuroscience Methods 134:9-21.

Sassenhagen J and Draschkow D (2019) Cluster-based permutation tests of MEG/EEG data do not establish significance of effect latency or location, Psychophysiology 56:e13335.
